# Focus on Translational Research from Arabidopsis to Crop Plants and Beyond

**DOI:** 10.1093/plcell/koaf119

**Published:** 2025-05-15

**Authors:** Adrienne H K Roeder, Cristiana T Argueso, Mary Williams, Gabriela Auge, Xin Li, Lucia Strader, Cristobal Uauy, Shuang Wu

**Affiliations:** School of Integrative Plant Science, Section of Plant Biology and Weill Institute for Cell and Molecular Biology, Cornell University, Ithaca, NY 14853, USA; Department of Agricultural Biology, Colorado State University, Fort Collins, CO 80523, USA; American Society of Plant Biologists, Rockville, MD 20855, USA; Consejo Nacional de Investigaciones Científicas y Tecnológicas (CONICET), Instituto de Agrobiotecnología y Biología Molecular (IABIMO), Instituto Nacional de Tecnología Agropecuaria (INTA)—CONICET, Hurlingham CP 1686, Argentina; Michael Smith Laboratories, University of British Columbia, Vancouver, BC, Canada, V6T 1Z4; Department of Biology, Duke University, Durham, NC 27708, USA; John Innes Centre, Norwich Research Park, Norwich NR4 7UH, UK; State Key Laboratory of Agricultural and Forestry Biosecurity, College of Horticulture, Fujian Agriculture and Forestry University, Fuzhou 350002, China

Over the past 4 decades, substantial research efforts in plant science worldwide have focused on the model system *Arabidopsis thaliana* (Provart et al. 2015). Many of us have dedicated years to the study of Arabidopsis, motivated by the notion that our findings will yield advancements in agriculture and natural ecosystems. Considering translation holistically as the gene functions, pathways, and technologies discovered and developed in Arabidopsis that inform our understanding of other plants, Arabidopsis has had a powerful influence. In particular, the annotation of plant genomes heavily relies on the gene functions elucidated in Arabidopsis (Whitt et al. 2020; Wimalanathan and Lawrence-Dill 2021; Fattel et al. 2022). Many technologies and techniques pioneered in Arabidopsis have been successfully translated to crops and other plant species (Yaschenko et al. 2025). Remarkably, discoveries in Arabidopsis and other plants have also contributed significantly to biomedical research (Jones et al. 2008; Strader et al. 2025). For example, the auxin degron system is widely used to degrade proteins on demand in animal/human cells by adding auxin to the cell cultures (Nishimura et al. 2009). Further, Arabidopsis research aimed at understanding plant adaptation to changing environments, with the goal of predicting evolutionary trajectories, holds the potential to guide conservation efforts in the face of climate change (Hancock et al. 2011; Assmann 2013; Wilczek et al. 2014; Arana and Picó 2025). However, we must ask the question of whether the features that make Arabidopsis a powerful model (i.e. its compact genome, ease of transformation, small size, and inexpensive growth) remain exclusive to this plant and whether the lessons learned from peculiar features of Arabidopsis can be extrapolated to plants we cultivate or those living in natural ecosystems, either directly or indirectly. Twenty-five years after the publication of the Arabidopsis genome sequence in 2000 (AGI 2000), it is a good time to reflect on the impact of research in Arabidopsis in this focus issue.

If we define translation strictly, in which a gene from Arabidopsis is expected to have a desired agronomic effect in the field when transformed into maize, the success rate stands at only 0.5% (Simmons et al. 2021 ). For comparison, only 2.0% of maize genes transformed into maize are successful in enhancing agronomic performance, indicating the challenges of this approach (Simmons et al. 2021). In conventional breeding, only a small percentage of lines that make it to yield trials will actually result in a released cultivar. Similarly, only 10% of drugs that enter clinical trials make it through to be used in human patients (Mullard 2016). On the other hand, there have been some spectacular translational successes. For example, CoverCress was developed through the rapid domestication of Pennycress (*Thlaspi arvense*, a close relative of Arabidopsis) as an oilseed winter cover crop based on knowledge from Arabidopsis (Chopra et al. 2018, 2020; Phippen et al. 2022, Basnet and Ellison 2024). Likewise, the PodGuard canola trait was developed by Bayer and is now widely distributed by BASF in North America based on fundamental work in Martin Yanofsky's laboratory on Arabidopsis fruit development and dehiscence (Liljegren et al. 2004; Liljegren et al. 2006 ; Vancanneyt et al. 2010; Aguilera 2019). These successes and the advent of genome editing technologies hint that a golden age of translation might be approaching in which the extensive knowledge of Arabidopsis can have creative applications in agriculture.

In this focus issue, we highlight breakthroughs in Arabidopsis research that have paved the way for discoveries and technology development in crops, in other plants, in non-model organisms, and in human health. We also underscore the limitations of this model, including constraints in translatability to crops due to differences in biology, genome organization, and field conditions. This focus issue includes 1 commentary, 1 timeline, 11 in-depth reviews, and at least 10 research papers. Further research papers will be included in the focus collection associated with this issue. The issue starts with a timeline of key milestones in Arabidopsis research, showcasing the development of new technologies, the growth of the Arabidopsis community, landmark papers published in *The Plant Cell*, essential research resources, and practical applications derived from Arabidopsis studies (Freed et al. 2025). This timeline is followed by a commentary defending the importance of funding fundamental plant science research in Arabidopsis and other plants as the foundation for innovations (Friesner et al. 2025).

Eleven reviews explore different aspects of how knowledge from Arabidopsis has and has not translated to other plants. First, Yaschenko et al. (2025) provide a comprehensive overview of Arabidopsis translational research. Next, we invited 7 experts in plant developmental biology to explore how developmental pathways that were elucidated in Arabidopsis apply to crop species (Bennett et al. 2025). Then, we gathered 7 experts on interactions with the abiotic environment to consider how findings in Arabidopsis have impacted research on crop resilience under stress (Roeder et al. 2025a). Bevan et al. (2025) review how a high degree of conservation of molecular mechanisms revealed by genome technologies bridges the gap between model and crop species, accelerating our understanding of the mechanisms underlying crop traits and improving their performance. Attallah et al. (2025) consider how work in model species laid the groundwork for applications of noncoding RNAs in agricultural biotechnology. Copeland et al. (2025) consider the extensive laboratory evidence for beneficial effects of the plant-associated microbiomes using Arabidopsis and other plants, as well as the challenges of translating this knowledge into practical applications in the field. Arana and Picó (2025) contemplate what researchers of other plant systems can learn from eco-evolutionary studies of Arabidopsis as well as what Arabidopsis researchers can learn from eco-evolutionary research in other plants. Sutherland et al. (2025) discuss how foundational knowledge on the biodiversity of plant immune receptors from studies on Arabidopsis and other plants has informed the ability to engineer immune receptors to confer a desired resistance. We brought together a group of scientists to consider the impacts of Arabidopsis research beyond plants on biomedical studies and human health, specifically innate immunity and repeat expansions in the genome as well as the development of pangenomics, synthetic biology parts for bioproduction, and the auxin inducible degron (Strader et al. 2025). We close out the issue with 2 more vignette style reviews. First, a group of experts consider challenges, but also successes, of translating discoveries from Arabidopsis into field-ready commercial cultivars (Uauy et al. 2025). Finally, another group of experts considers attributes of Arabidopsis that have been “lost in translation,” such as floral dip transformation, direct gene orthology, arbuscular mycorrhizal symbiosis, specialized cell types, and C_4_ photosynthesis, among others (Roeder et al. 2025b). Despite such losses, 1 theme that emerges is that insights can still be gained from comparisons with Arabidopsis.

The research papers in this focus issue reveal fundamental insights into how discoveries and technologies traverse across Arabidopsis and other plant systems. First, the *SEPALLATA* genes control floral organ identity in Arabidopsis (Pelaz et al. 2000; Ditta et al. 2004), whereas Song et al. (2024) found that a natural polymorphism of *SEPALLATA2* in cucumber controls fruit length. Continuing the floral organ identity theme, building on the ABC model of floral organ identity developed in Arabidopsis and snapdragon (Coen and Meyerowitz 1991 ), Yang et al. (2024) found that a MITE element had inserted into the promoter of a B class gene *GLOBOSA1* causes ectopic expression in all floral whorls, creating a hose-in-hose phenotype with 2 tubes of petals in *Sinningia*. Turning to light signaling, Kunta et al. (2025) compare the roles of PHYTOCHROME-INTERACTING FACTORs (PIFs) in tomato with functions that have been established in Arabidopsis, finding that *SlPIF8a* plays an important role in red/far red light response in tomato whereas the same function is regulated primarily by *PIF7* in Arabidopsis, with no role for *PIF8* in shade avoidance. Continuing the theme of growth control, Sun et al. (2025) dissect the tail of Gγ proteins, which they show provide specificity to signaling, controlling rice grain length. They compare with an Arabidopsis Gγ tail in their evolutionary analysis noting that both Arabidopsis and maize G protein signaling also regulate organ size. Nutrients are also essential for plant growth, and using fine time-resolved RNA-seq data sets in both Arabidopsis and maize, Huang et al. (2025a) develop model-to-crop gene regulatory network regulons of importance to nitrogen use efficiency. They next used machine learning to predict nitrogen use efficiency traits in maize and Arabidopsis.

In a great example of translation of Arabidopsis knowledge to crops, Castro et al. (2025) used previous discoveries about the regulation of ROS production in Arabidopsis to generate tomato plants that are more resistant to pathogens. The researchers edited a specific amino acid residue of the tomato *P*bl13 *i*nteracting *R*ING domain *E*3 ligase (PIRE), which in Arabidopsis regulates RBOH protein stability and ROS levels important for defense. Just like in Arabidopsis, the edited version containing the specific mutation led to increased RBOH protein levels and broad-spectrum defense against foliar pathogens. An intriguing finding was that this editing of *PIRE* in tomato did not provide resistance to diseases caused by root pathogens, which increases its agricultural applicability as it can be potentially used to control foliar diseases without disrupting plant interactions with root-colonizing beneficial microbes (Castro et al. 2025).

Several other crops studies benefitted from knowledge previously gained in Arabidopsis to improve important agronomic traits: Armed with knowledge from Arabidopsis auxin signaling, Liu et al. (2025) showed that in cucumber seeds, orthologues of ARF9 and FUSCA opposingly regulate sugar metabolism by transcriptional regulation of a gene encoding an alkaline a-galactosidase, altering seed filling and therefore also final seed size, with important consequences to seed yield for breeding purposes. In strawberries, comprehension of the COP signalosome characterized in Arabidopsis led Huang et al. (2025b) to demonstrate that levels of polyamines are regulated by proteasome-induced degradation of *POLYAMINE OXIDASE 5*, changing the levels of polyamines available for growth or ROS scavenging, increasing fruit growth and changing fruit ripening, including in commercialized polyploid varieties. And building on previous studies of brassinosteroid signaling elucidated in Arabidopsis, Liu et al. (2024) showed that the rice R2R3 MYB transcription factor FOUR LIPS links BR signaling and lignin deposition at the lamina joint, which is essential for the final leaf angle, an important trait for plant architecture, light capturing, and ultimately yield.

A large part of the studies highlighted here are directly linked to the signaling and metabolism of phytohormones, which, as main regulators of plant development and responses to the environment, tend to regulate important agronomic traits. And while the canonical signaling pathways of these phytohormones tend to be conserved between Arabidopsis and crops, differences in their regulation can be important for how traits are manipulated in translational efforts. As an example of differential regulation, Chen et al. (2024) integrated transcriptomics, ChIP-seq, and metabolomics data of different rice tissues after treatment with jasmonic acid and demonstrated that the core transcriptional response to JA is regulated by the rice orthologue of MYC2, a well-known regulator of JA responses in Arabidopsis. However, their detailed analyses identified tissue-specificity of certain subnetworks, unknown in Arabidopsis, some of which are important for defense against herbivores and for repression of JA signaling to allow for plant growth, differently modulating growth and defense in distinct tissues of the rice plant. This paper illustrates the theme that tissue-specific or fine-tuned hormone signaling appears to be a key strategy for optimizing crop traits.

Collectively, these articles showcase the ongoing importance of Arabidopsis in elucidating fundamental plant biology that informs research on crops and other plants, as well as biological systems in general. It also illustrates the tight web of information flow in all directions, with work in crop plants advancing understanding in Arabidopsis. While many mechanisms, at least conceptually, can translate between plants, the articles also highlight the challenges of going from basic discovery to field-ready commercial cultivars. And finally, it is worth investigating all plants which have uniquely adaptive and specialized mechanisms that may not be translated to other plants, but for which the comparison of how they do not translate can inform the mechanism. We encourage authors to continue to submit their best work in this area to *The Plant Cell*, and articles published in the next 8 to 12 mo will continue to be added to the online focus collection on Translational Research from Arabidopsis to Crop Plants and Beyond.

## Data Availability

No new data were generated or analysed in support of this editorial.
